# Supplementary material for: Microglial neuropilin-1 promotes oligodendrocyte expansion during development and remyelination by trans-activating platelet-derived growth factor receptor
Source: Nat Commun. 2021 Apr 15;12:2265. doi: 10.1038/s41467-021-22532-2 (PMC8050320; doi:10.1038/s41467-021-22532-2)
Supplement: Supplementary file 1 — Supplementary Information [file 41467_2021_22532_MOESM1_ESM.pdf]

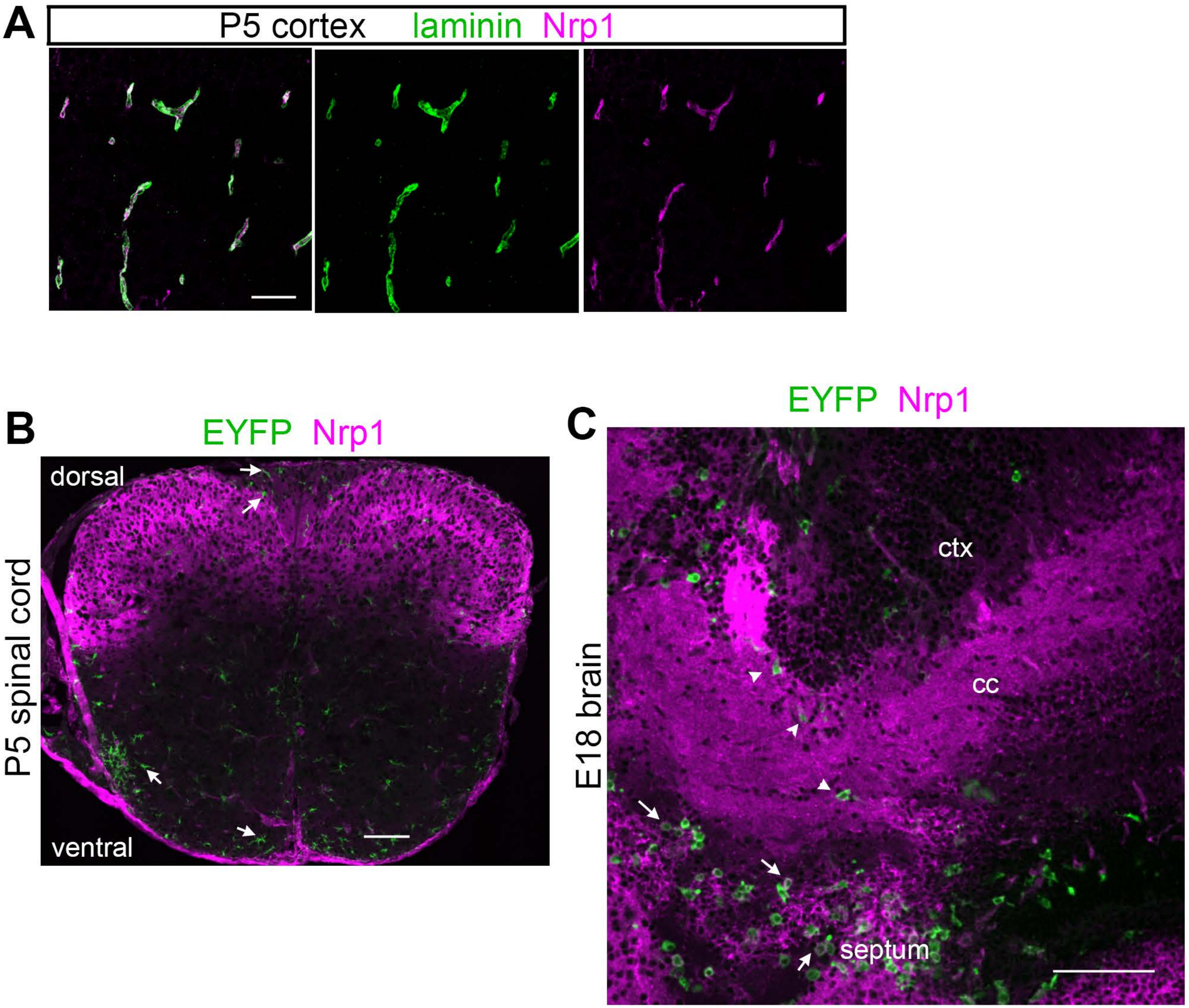

Sherafat et al., Supplemental Figure 1

Supplementary Fig 1. The distribution of Nrp1 in the developing CNS

A. Nrp1 expression on laminin+ blood vessels in P5 cortex. Green, laminin; magenta, Nrp1. Scale, 20 μm.

B. P5 thoracic spinal cord from Cx3CR1$^{creERT2\text{-}ires\text{-}EYFP}$ mouse stained for Nrp1 (magenta). EYFP+ microglia in the white matter are Nrp1-negative (arrows). Strong neuronal Nrp1 expression is seen in the dorsal spinal cord. Scale, 50 μm.

C. Coronal section of forebrain from E18.5 Cx3CR1$^{creERT2\text{-}ires\text{-}EYFP}$ mouse showing Nrp1 (magenta) on axons in the dorsal corpus callosum. YFP+ microglia in the corpus callosum do not express Nrp1 (arrowheads), while the round YFP+ meningeal macrophage-like cells near the septum are Nrp1+ (arrows). Scale, 50 μm. ctx, cortex; cc, corpus callosum.

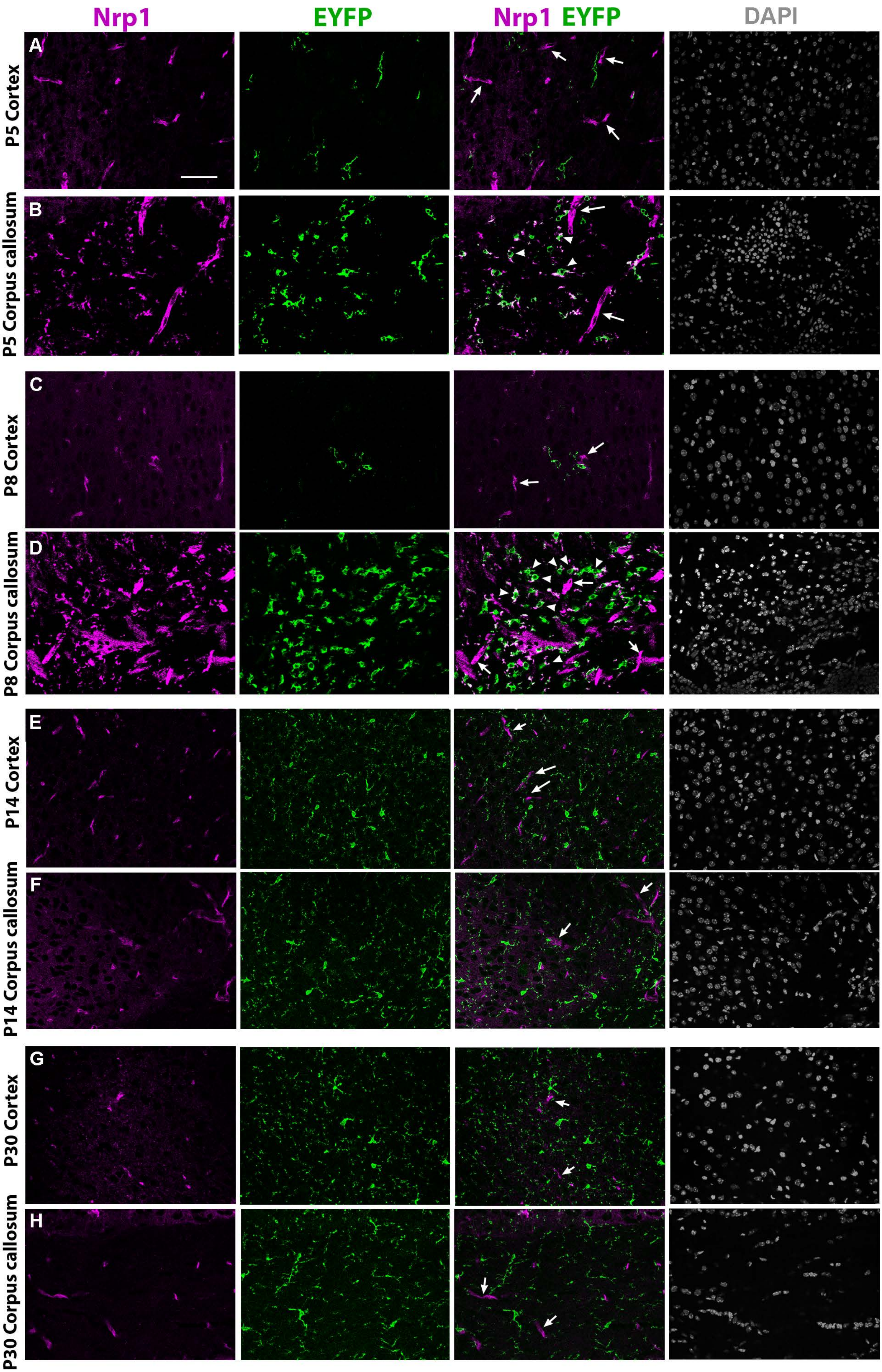

Sherafat et al., Supplemental Figure 2

Supplementary Fig 2. Nrp1 expression on EYFP+ microglia in the cortex and corpus callosum during postnatal development of Cx3CR1$^{creERT2\text{-}ires\text{-}EYFP}$ mice.

A. P5 cortex

B. P5 corpus callosum

C. P8 cortex

D. P8 corpus callosum

E. P14 cortex

F. P14 corpus callosum

G. P30 cortex

H. P30 corpus callosum

Npr1, magenta; EYFP, green. DAPI, gray. Arrows, examples of blood vessels. Arrowheads, examples of Nrp1+ EYFP+ microglia. Scale bar in A = 50 μm.

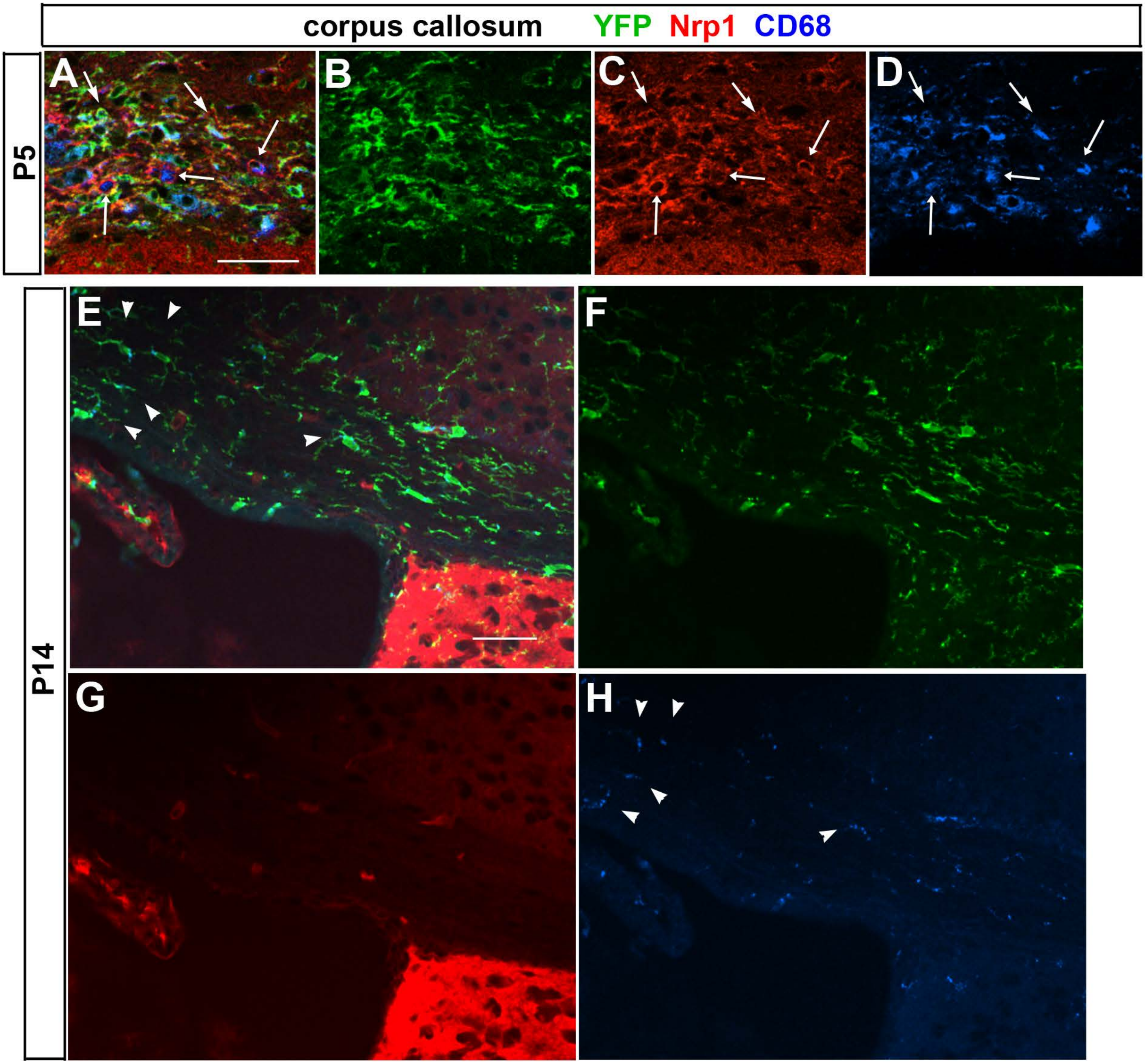

Sherafat et al., Figure S2

Supplementary Fig 3. CD68 is expressed on Nrp1+ amoeboid microglia.

A-D. CD68 is detected in some Nrp1+ amoeboid microglia in P5 corpus callosum (arrows). Scale, 50 μm.

E-H. In P14 corpus callosum, Nrp1 is no longer detected on microglia that appear ramified. These microglia have significantly less CD68 immunoreactivity than those in P5 corpus callosum, and it is restricted a few puncta (arrowheads). Scale, 50 μm.

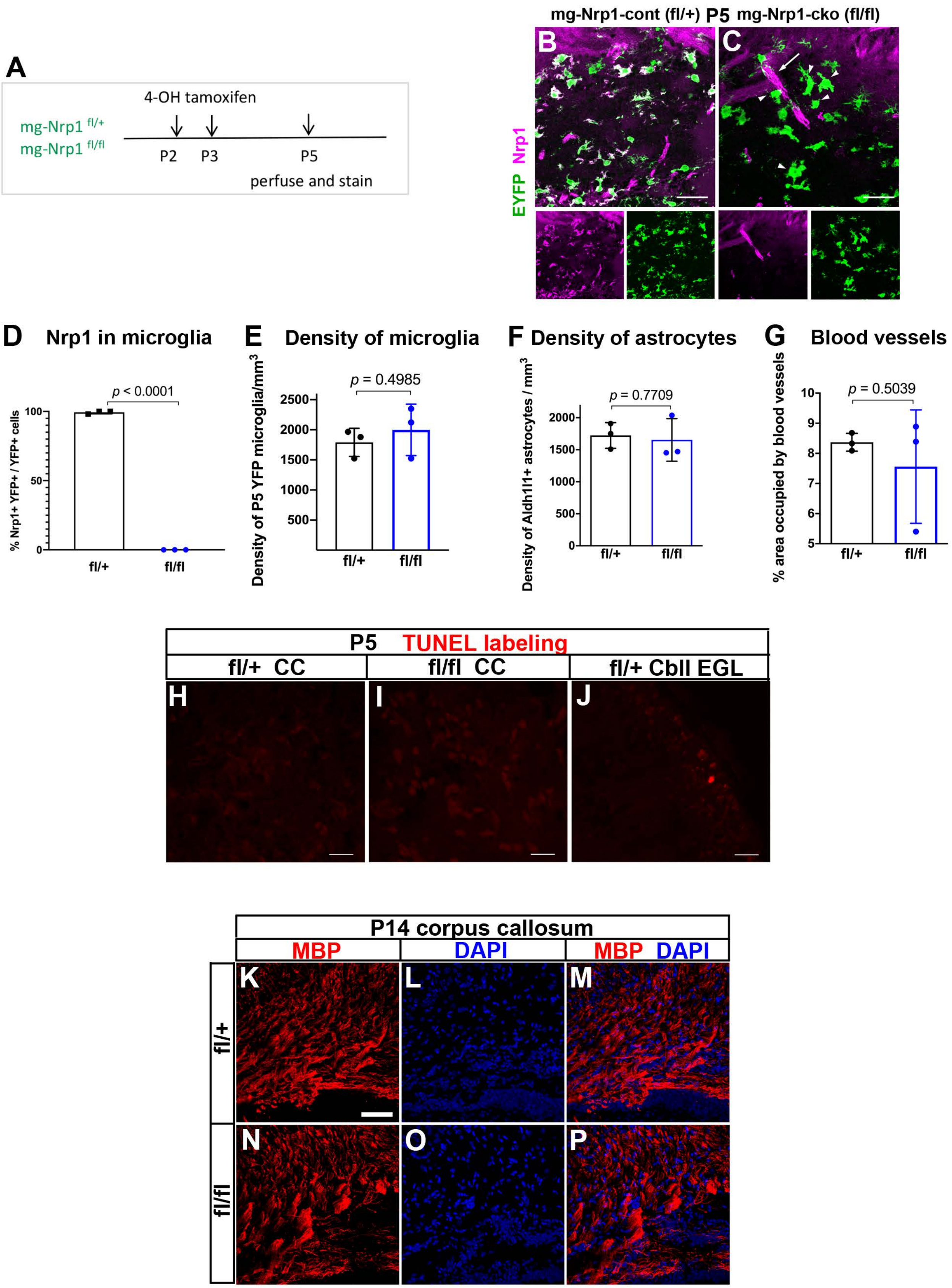

Sherafat et al., Supplemental Figure 4

Supplementary Fig 4. Characterization of mg-Nrp1-cko.

A. Schematic of generating microglia-specific Nrp1 knock out (mg-Nrp1-cko) and the heterozygous control (mg-Nrp1-cont).

B-C. P5 corpus callosum after 4OHT injection at P2-3, showing a complete absence of Nrp1 on EYFP+ microglia (arrowheads show examples) but retained on blood vessels (arrow). Scale, 50 μm.

D. The proportion of Nrp1+ EYFP+ cells among EYFP+ microglia in the corpus callosum of P5 mg-Nrp1-cont and mg-Nrp1-cko mice. Student's t-test, n = 3, t = 156.9, df =4.

E. The density of Nrp1+ EYPF+ microglia in the corpus callosum of P5 mg-Nrp1-cont and mg-Nrp1-cko mice. Student's t-test, n = 3, t = 0.7434, df =4.

F. The density of Aldh1L1+ astrocytes in the corpus callosum of P5 mg-Nrp1-cont and mg-Nrp1-cko mice. Student's t-test, n = 3, t = 0.3116, df =4.

G The proportion of the corpus callosum area occupied by blood vessels outlined by laminin immunoreactivity. Student's t-test, n = 3, t = 0.5039, df =4.

H-J. TUNEL labeling of P5 corpus callosum of mg-Nrp1-cont (**F**) and mg-Nrp1-cko (**G**) and the external granule cell layer of the cerebellar cortex of mg-Nrp1-cont, showing a positive cell (**H**). Scale 20 μm.

K-P. Immunolabeling for MBP in the corpus callosum of P14 mg-Nrp1-cont and cko mice. Scale 50 μm.

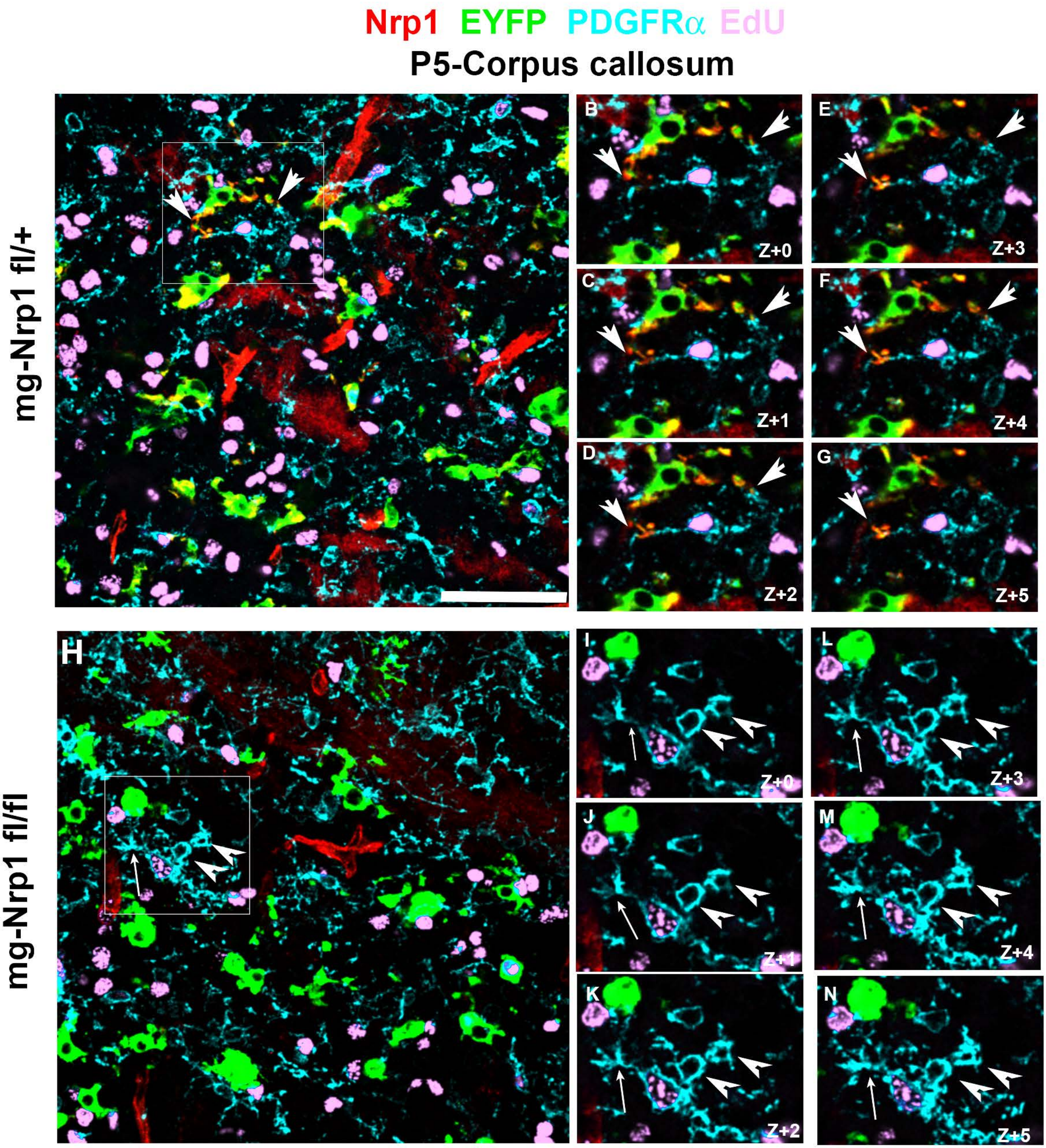

Sherafat et al., Supplemental Figure 5

Supplementary Fig 5. Examples of contact between proliferating OPCs and microglia in P5 corpus callosum

A. Maximum projection of 8 *z*-slices, 35 μm apart, from mg-Nrp1-cont labeled for Nrp1, EYFP, PDGFRα, and EdU. Scale, 50 μm. Scale, 50 μm.

B-G. A series of selected individual consecutive *z*-sections from the boxed area in A. *z*=0.35μm. Two processes of an EdU+ PDGFRα+ OPC have contacts (short arrows) with Nrp1+ processes of an EYFP+ microglia above the OPC.

H. Maximum projection of 8 *z*-slices, 35 μm apart, from mg-Nrp1-cko corpus callosum labeled for Nrp1, EYFP, PDGFRα, and EdU. Nrp1 is deleted from EYFP+ microglia, while it is retained on blood vessels.

I-N. A series of consecutive *z*-sections from the boxed area in H. *z*=0.35μm.

Two EdU-negative PDGFRα+ OPCs do not appear to have contact with the adjacent EYFP+ microglia (arrowheads). The distal process of on EdU+ PDGFRα+ OPC immediately to the left may be contacting the EYFP+ Nrp1-negative microglia above (long arrows).

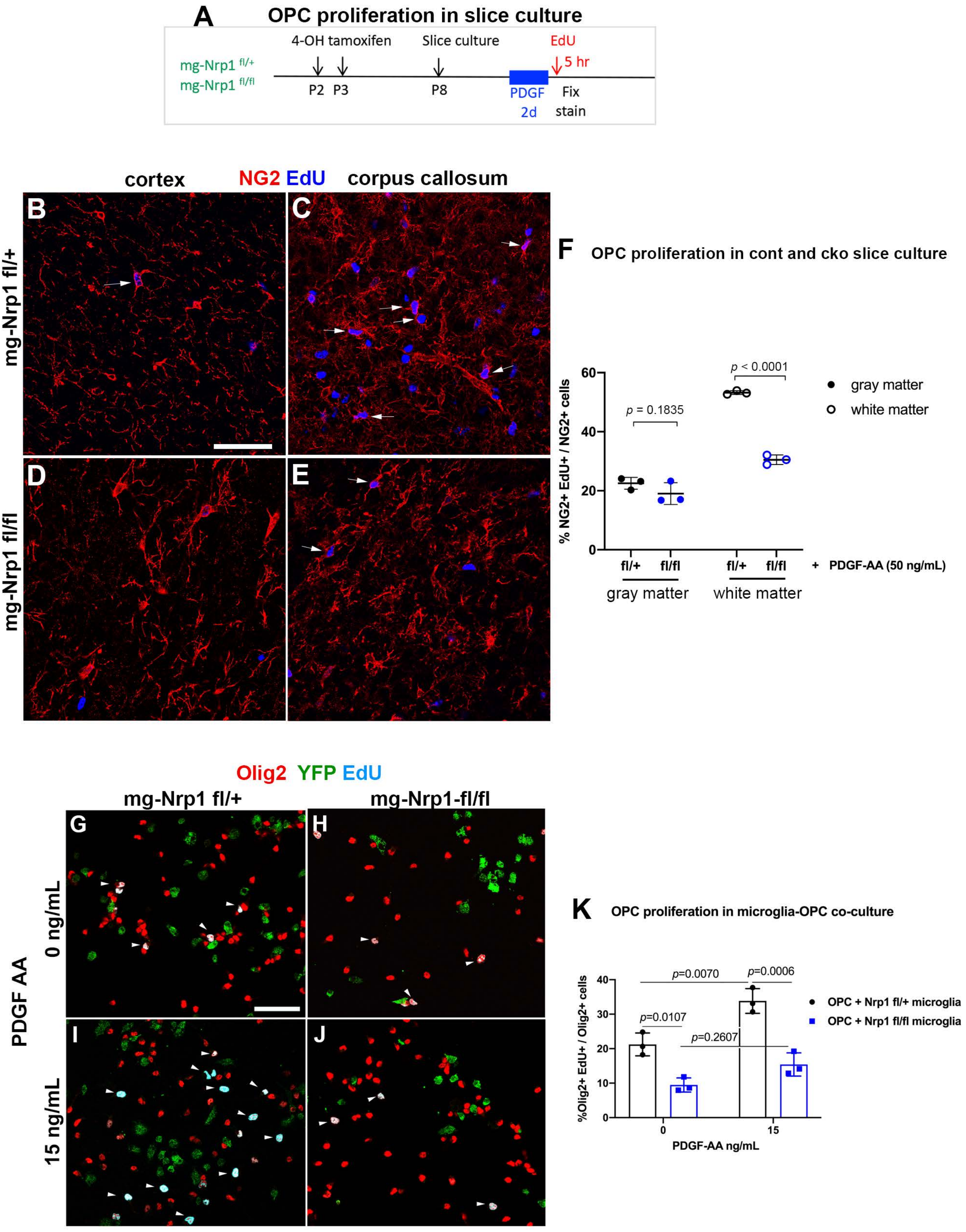

Sherafat et al., Supplemental Figure 6

Supplementary Fig 6. PDGF AA-mediated OPC proliferation in mg-Nrp1-cont and cko in slice cultures and dissociated cultures.

A. Schematic showing EdU labeling of slice cultures from P8 mg-Nrp1-cont or cko mice after cre induction in vivo at P2 and P3.

B-E. EdU incorporation into NG2+ OPCs in mg-Nrp1 cont cortex (B) and corpus callosum (C) and mg-Nrp1-cko cortex (D) and corpus callosum (E). Scale in B, 50 μm. Arrows, EdU+ NG2+ OPCs.

F. Quantification showing the proportion of NG2+ cells that were EdU+ in PDGF AA-treated slices. Two-way ANOVA, Tukey's multiple comparisons test, n=3, $F(1, 8) = 100.4$.

G-J. OPCs cocultured with microglia from mg-Nrp1-cont (G, I) or mg-Nrp1-cko (H, J) mice in the presence of 0 (G, H) or 15 ng/mL PDGF AA (I, J) and labeled for Olig2, YFP, and EdU. Scale in B, 50 μm. Arrowhead, EdU+ OPCs.

Proliferation of OPCs cocultured with microglia from mg-Nrp1-cont (black) or mg-Nrp1-cko (blue) mice in the presence of 0 or 15 ng/mL PDGF AA. Two-way ANOVA, Sidak's multiple comparisons test. Comparison between cont and cko: n = 3, $F(1, 8) = 26.17$. Comparison between 0 and 15 ng/mL PDGF AA: n = 3, $F(1,8) = 69.19$.

K. Proliferation of OPCs cocultured with microglia from mg-Nrp1-cont (black) or mg-Nrp1-cko (blue) mice in the presence of 0 or 15 ng/mL PDGF AA. Two-way ANOVA, Sidak's multiple comparisons test. Comparison between cont and cko: n = 3, $F(1, 8) = 26.17$. Comparison between 0 and 15 ng/mL PDGF AA: n = 3, $F(1,8) = 69.19$.

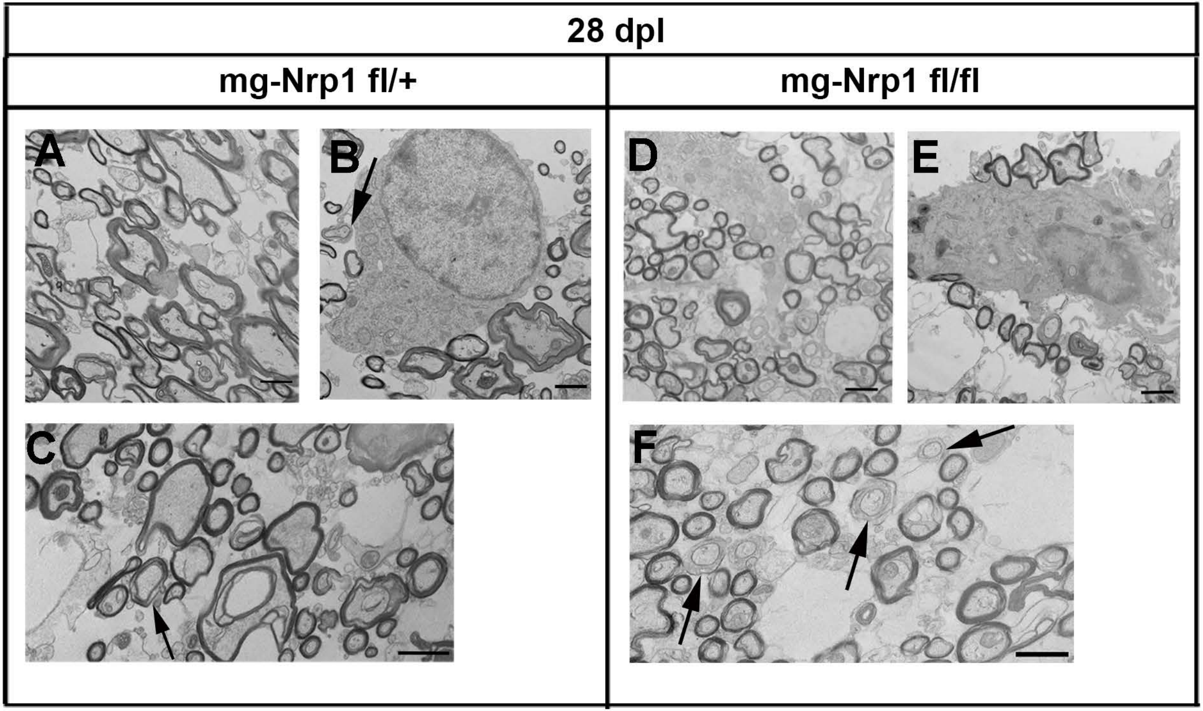

Sherafat et al., Supplemental Figure 7

Supplementary Fig 7. Repair processes at 28 dpl in mg-Nrp1-cont and cko. Electron microscopic images of cross-sections of LPC-lesioned corpus callosum at 28 dpl.

A-C: mg-Nrp1-cont (fl/+) show occasional glial processes (**A**) including a typical oligodendrocyte (**B**) surrounded by a few myelinated axons and one that still appears to be in the process of myelination (arrow in **B**). Occasional axons are surrounded by non-compacted myelin (**C**, arrow).

D-F: mg-Nr1p-cko (fl/fl) contain more glial processes and glial cells near axons with various extent of myelination / ensheathment (**D**). A typical oligodendrocyte surrounded by axons with varying thickness of myelin sheaths (**E**). Axons surrounded by non-compacted protrusions of glial cell processes are frequent (**F**, arrows).

Scale, 1 μm.

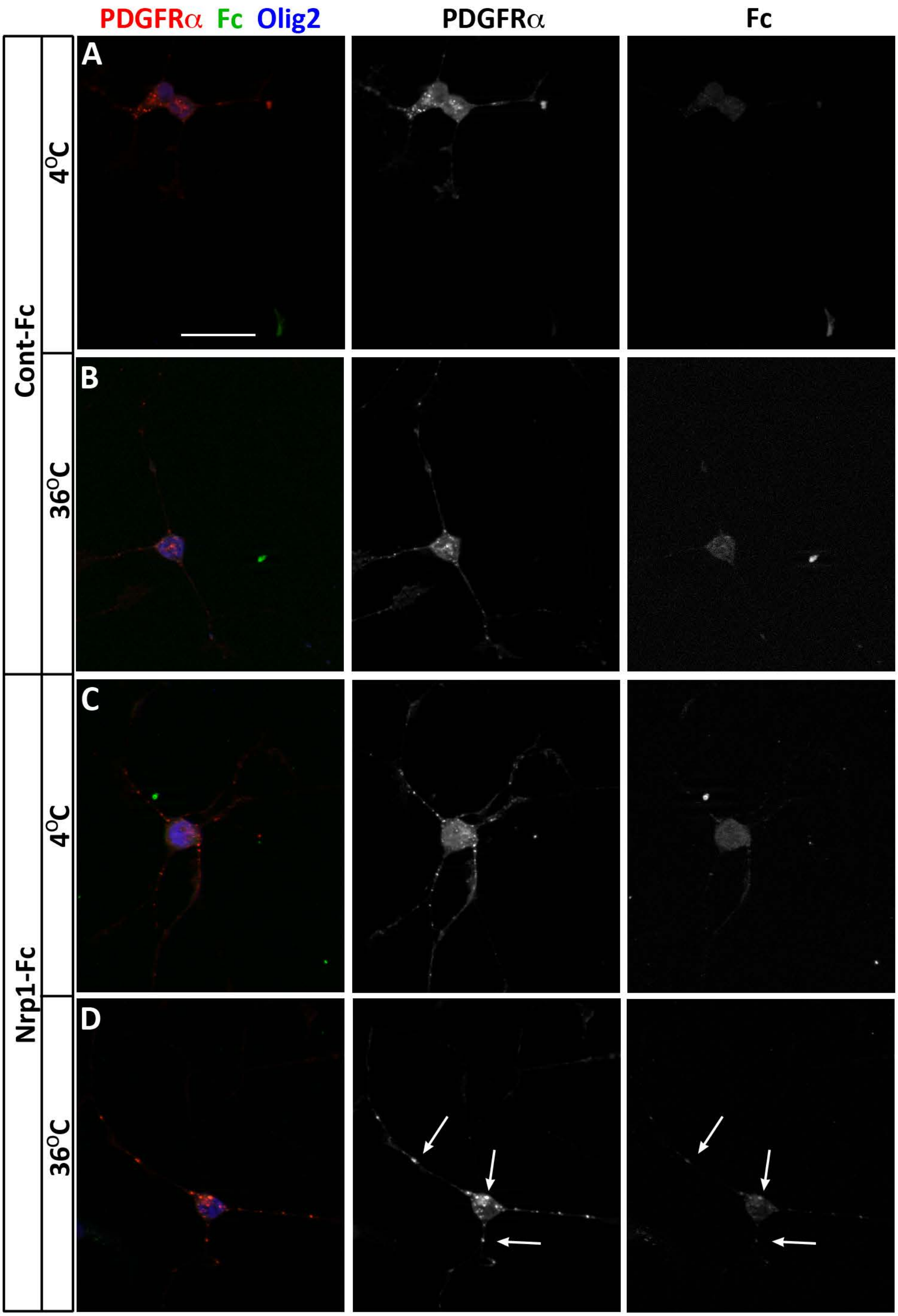

Sherafat et al., Supplemental Figure 8

Supplementary Fig 8. Co-clustering of Nrp1-Fc with PDGFRα on dissociated OPCs.

A-B. Immunopanned OPCs were incubated for 30 minutes with control human IgG Fc dimer (Cont-Fc) at 4°C (A) or 36°C (B) and stained for PDGFRα and Fc.

C-D. Immunopanned OPCs were incubated for 30 minutes with Nrp1-Fc fusion protein (Nrp1-Fc) at 4°C (A) or 36°C (B) and stained for PDGFRα and Fc.

Arrows show Nrp1-Fc co-clustered with PDGFRα. Scale, 20 μm.

Supplementary Table 1. PCR Primers used for mouse genotyping

Name	Forward sequence	Reverse sequence 1	Reverse sequence 2
Cx3Cr1 cre $^{ERT2:YFP}$	5' - AAG ACT CAC GTG GAC CTG CT	5' - CGG TTA TTC AAC TTG CAC CA	5' - AGG ATG TTG ACT TCC GAG TTG
Nrp1	5' - AGG TTA GGC TTC AGG CCA AT	5' - GGT ACC CTG GGT TTT CGA TT	
Cre	5' - CCG TAC ACC AAA ATT TGC C	5' - ATC GCG AAC ATC TTC AGG	
