# Supplementary material for: Microglial neuropilin-1 promotes oligodendrocyte expansion during development and remyelination by trans-activating platelet-derived growth factor receptor
Source: Nat Commun. 2021 Apr 15;12:2265. doi: 10.1038/s41467-021-22532-2 (PMC8050320; doi:10.1038/s41467-021-22532-2)
Supplement: Supplementary file 3 — Reporting Summary [file 41467_2021_22532_MOESM3_ESM.pdf]

nature research

Corresponding author(s): Akiko Nishiyama

Last updated by author(s): 2/23/2021

Reporting Summary

Nature Research wishes to improve the reproducibility of the work that we publish. This form provides structure for consistency and transparency in reporting. For further information on Nature Research policies, see Authors & Referees and the Editorial Policy Checklist .

Statistics

For all statistical analyses, confirm that the following items are present in the figure legend, table legend, main text, or Methods section.

n/a	Confirmed	
☐	☒	The exact sample size (*n*) for each experimental group/condition, given as a discrete number and unit of measurement
☒	☐	A statement on whether measurements were taken from distinct samples or whether the same sample was measured repeatedly
☐	☒	The statistical test(s) used AND whether they are one- or two-sided<br>*Only common tests should be described solely by name; describe more complex techniques in the Methods section.*
☒	☐	A description of all covariates tested
☐	☒	A description of any assumptions or corrections, such as tests of normality and adjustment for multiple comparisons
☐	☒	A full description of the statistical parameters including central tendency (e.g. means) or other basic estimates (e.g. regression coefficient) AND variation (e.g. standard deviation) or associated estimates of uncertainty (e.g. confidence intervals)
☐	☒	For null hypothesis testing, the test statistic (e.g. *F*, *t*, *r*) with confidence intervals, effect sizes, degrees of freedom and *P* value noted<br>*Give P values as exact values whenever suitable.*
☒	☐	For Bayesian analysis, information on the choice of priors and Markov chain Monte Carlo settings
☒	☐	For hierarchical and complex designs, identification of the appropriate level for tests and full reporting of outcomes
☒	☐	Estimates of effect sizes (e.g. Cohen's *d*, Pearson's *r*), indicating how they were calculated

Our web collection on statistics for biologists contains articles on many of the points above.

Software and code

Policy information about availability of computer code

Data collection	image acquisition: 1) Leica SP8 TCS Spectral Confocal Microscope with LAX software; 2) Zeiss Axiovert M200 with Axiovision software<br>Immunoblotting: LI-COR Odyssey
Data analysis	Image analysis: Leica LAS X v3.3.0 and Zeiss Axiovision v4.8; Quantification on images: Leica LAS X v3.3.0 and ImageJ v1.53a; generation of figures: AdobePhotoshop CS5 and CC2018, 2020<br>Immunoblotting: ImageStudioLite v5.2.5<br>Analysis of quantification data and generation of graphs: GraphPad Prism v8 and v9.

For manuscripts utilizing custom algorithms or software that are central to the research but not yet described in published literature, software must be made available to editors/reviewers. We strongly encourage code deposition in a community repository (e.g. GitHub). See the Nature Research guidelines for submitting code & software for further information.

Data

Policy information about availability of data

All manuscripts must include a data availability statement. This statement should provide the following information, where applicable:

- Accession codes, unique identifiers, or web links for publicly available datasets
- A list of figures that have associated raw data
- A description of any restrictions on data availability

The datasets generated and analyzed in this study are available from the corresponding author upon reasonable request.

Field-specific reporting

Please select the one below that is the best fit for your research. If you are not sure, read the appropriate sections before making your selection.

☒ Life sciences ☐ Behavioural & social sciences ☐ Ecological, evolutionary & environmental sciences

For a reference copy of the document with all sections, see nature.com/documents/nr-reporting-summary-flat.pdf

Life sciences study design

All studies must disclose on these points even when the disclosure is negative.

Sample size	The sample sizes for tissue analyses were determined based on the practice in the field, the variability obtained from pilot studies, and with the aim to reduce redundancy (3R rule according to IACUC regulations) while providing sufficient power to detect the differences between control and experimental groups. The sample sizes for slice and dissociated culture analyses were determined based on the practice in the field, the variability obtained from pilot studies. The sample size was sufficient because the mean differences between the groups were sufficiently large to allow us to detect differences.
Data exclusions	We did not exclude any data.
Replication	For tissue analyses, mice were obtained from more than two different litters, and experiments were performed 3 times for developmental studies and 5 times for lesioning studies.<br>For slice cultures, replicates were obtained from different mice, and experiments were performed three times.<br>For dissociated cell cultures, replicates were obtained from different litters of pups, and experiments were performed three times.<br>All replicates yielded similar results.
Randomization	For all the experiments, an appropriate number of mice of each genotype were randomly selected from one or multiple litters of mice. Both males and females were used.
Blinding	Investigators were not blinded during data collection and analysis because we did not have a sufficient number of people to perform blinded studies. Data collection were performed to eliminate bias by randomly capturing image fields based on DAPI channel and then opening the other channels for quantification. Quantification results were cross-checked by the corresponding author against the raw data, i.e. original microscope slides and blots.

Reporting for specific materials, systems and methods

We require information from authors about some types of materials, experimental systems and methods used in many studies. Here, indicate whether each material, system or method listed is relevant to your study. If you are not sure if a list item applies to your research, read the appropriate section before selecting a response.

Materials & experimental systems

n/a	Involved in the study	
☐	☒	Antibodies
☒	☐	Eukaryotic cell lines
☒	☐	Palaeontology
☐	☒	Animals and other organisms
☒	☐	Human research participants
☒	☐	Clinical data

Methods

n/a	Involved in the study	
☒	☐	ChIP-seq
☒	☐	Flow cytometry
☒	☐	MRI-based neuroimaging

Antibodies

Antibodies used	Please see Table 1 for all primary and secondary antibodies used in the staining for immunofluorescence labeling, immunoblotting, and immunopanning. The rabbit PDGFRa antibody was a gift from Dr. William Stallcup (Sanford-Burnham Medical Research Institute)
Validation	RRIDs are given for each primary antibody. All primary antibodies were validated either by the manufacturer or by the investigator who had donated the antibodies. Rabbit anti-rat PDGFRa antibody supplied by Dr. William Stallcup has been verified against null mutant mouse tissue. Detailed in the methods.

Animals and other organisms

Policy information about studies involving animals; ARRIVE guidelines recommended for reporting animal research

Laboratory animals	Strains of mice used: 1) Nrp1-fl (Jackson Laboratory stock no. 005247, B6.129(SJL)-Nrp1tm2Ddg/J; RRID:MGI:3528190); 2) Cx3cr1creERT2-ires-EGFP (Jackson Laboratory stock no. 021160 B6.129P2(Cg)-Cx3cr1tm2.1(cre/ERT2)Litt/WganJ; RRID:MGI:5528845); 3) Z/EG (Jackson Laboratory stock no. 003920, STOCK Tg(CAG-Bgeo/GFP)21Lbe/J; RRID:IMSR_JAX:003920); 4) NG2cre (NG2creERA BAC transgenic mice (described in Zhu et al., 2011)<br>Both males and females were used for all experiments.<br>Mice were housed in a facility with 12:12 light:dark cycle, 50% humidity, and 73.5oC.
Wild animals	none
Field-collected samples	none
Ethics oversight	University of Connecticut Institutional Animal Care and Use Committee

Note that full information on the approval of the study protocol must also be provided in the manuscript.
